# 541 Granzyme B: Potential Role in Hypertrophic Scarring and Burn Wound Healing Through Extracellular Matrix Remodeling

**DOI:** 10.1093/jbcr/iraf019.170

**Published:** 2025-04-01

**Authors:** Alexandre Aubert, David Granville

**Affiliations:** University of British Columbia; University of British Columbia

## Abstract

**Introduction:**

Hypertrophic scars are fibroproliferative wound healing defects affecting up to 70% of patients with severe burns. Characterized by extracellular matrix (ECM) accumulation in the dermis, hypertrophic scars are often associated with an excessive production and activation of transforming growth factor (TGF)-Beta, a major pro-fibrotic cytokine. Granzyme B (GzmB) is a serine protease that is elevated in burn wounds and may contribute to scarring and fibrosis through the degradation of specific ECM proteins. GzmB notably cleaves Decorin, leading to the impairment of collagen fibrillogenesis and the subsequent release/activation of TGF-Beta. Furthermore, GzmB topical inhibition reduced scarring and decorin loss in a mouse model of burn wound healing. The present study aims at delineating the role of GzmB in hypertrophic scarring through the cleavage of the ECM, with a specific focus on TGF-Beta activation.

**Methods:**

Using skin sections from healthy controls (n=6) and patients with hypertrophic scars (n=10), we investigated GzmB ability to regulate TGF-Beta activation through the cleavage of extracellular matrix proteins.

**Results:**

Immunohistochemical staining and co-immunofluorescence analyses showed an accumulation of GzmB-positive cells, especially mast cells, in the dermis of hypertrophic scar (n=10) patients compared to healthy controls (n=6). Staining for substance P, a neuropeptide involved in mast cell degranulation, suggests that GzmB is released extracellularly by mast cells. Compared to healthy skin, we confirmed the reduction of decorin and observed a loss of the ECM protein LTBP1 (Latent TGF-Beta Binding Protein-1) at the dermal-epidermal junction of hypertrophic scars. Using LTBP1 producing cells, its cleavage was confirmed by GzmB in vitro. Furthermore, increased activation of the TGF-beta/Smad signaling pathway was observed in cells treated with LTBP1 pre-digested with GzmB. This observation, abolished by a Pan-TGF-Beta inhibitor, indicates that GzmB promotes LTBP1 dependent TGF-Beta activation.

**Conclusions:**

GzmB may contribute to hypertrophic scar formation through multiple mechanisms involving ECM cleavage and TGF-Beta activation.

**Applicability of Research to Practice:**

This study will provide key rationale to pursue GzmB as a novel therapeutic target for the treatment of hypertrophic scars.

**Funding for the Study:**

This work was funded in part by grants-in-Aid from the Canadian Institutes for Health Research, National Sciences and Engineering Research Council of Canada, the British Columbia Professional Firefighters’ Burn Fund, and the International Brotherhood of Electrical Workers (IBEW) Local 258. One of the author is the recipient of the Arthritis Society Canada Training Postdoctoral Fellowship.

